# Vitronectin‐activated αvβ3 and αvβ5 integrin signalling specifies haematopoietic fate in human pluripotent stem cells

**DOI:** 10.1111/cpr.13012

**Published:** 2021-03-03

**Authors:** Jun Shen, Yaoyao Zhu, Shuo Zhang, Shuzhen Lyu, Cuicui Lyu, Zicen Feng, Dixie L. Hoyle, Zack Z. Wang, Tao Cheng

**Affiliations:** ^1^ State Key Laboratory of Experimental Hematology, National Clinical Research Center for Blood Diseases, Institute of Hematology & Blood Diseases Hospital Chinese Academy of Medical Science & Peking Union Medical College Tianjin China; ^2^ Department of Hematology the First Central Hospital of Tianjin Tianjin China; ^3^ Center of Reproductive Medicine Tianjin Central Hospital of Gynaecology and Obstetrics Tianjin China; ^4^ Division of Hematology Johns Hopkins University School of Medicine Baltimore MD USA; ^5^ Department of Stem Cell and Regenerative Medicine Peking Union Medical College Tianjin China; ^6^ Tianjin Key Laboratory of Blood Cell Therapy and Technology Tianjin China

**Keywords:** extracellular matrix, haematopoietic differentiation, human pluripotent stem cells, integrin, vitronectin

## Abstract

**Objectives:**

Vitronectin (VTN) has been widely used for the maintenance and expansion of human pluripotent stem cells (hPSCs) as feeder‐free conditions. However, the effect of VTN on hPSC differentiation remains unclear. Here, we investigated the role of VTN in early haematopoietic development of hPSCs.

**Materials and Methods:**

A chemically defined monolayer system was applied to study the role of different matrix or basement membrane proteins in haematopoietic development of hPSCs. The role of integrin signalling in VTN‐mediated haematopoietic differentiation was investigated by integrin antagonists. Finally, small interfering RNA was used to knock down integrin gene expression in differentiated cells.

**Results:**

We found that the haematopoietic differentiation of hPSCs on VTN was far more efficient than that on Matrigel that is also often used for hPSC culture. VTN promoted the fate determination of endothelial‐haematopoietic lineage during mesoderm development to generate haemogenic endothelium (HE). Moreover, we demonstrated that the signals through αvβ3 and αvβ5 integrins were required for VTN‐promoted haematopoietic differentiation. Blocking αvβ3 and αvβ5 integrins by the integrin antagonists impaired the development of HE, but not endothelial‐to‐haematopoietic transition (EHT). Finally, both αvβ3 and αvβ5 were confirmed acting synergistically for early haematopoietic differentiation by knockdown the expression of αv, β3 or β5.

**Conclusion:**

The established VTN‐based monolayer system of haematopoietic differentiation of hPSCs presents a valuable platform for further investigating niche signals involved in human haematopoietic development.

## Graphical abstract

Compared with Matrigel (MTG), vitronectin (VTN) was required for the mesoderm to acquire higher endothelial‐hematopoietic potential. The promoting effect of VTN on early hematopoiesis was dependent on αvβ3 and αvβ5 integrins. Inhibition of αvβ3 and αvβ5 impaired HE development without affecting EHT.

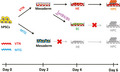

## 1 INTRODUCTION

Human embryonic haematopoiesis is a complex‐regulated process with multiple developmental steps, including mesodermal induction, the development of endothelial progenitors and endothelial‐to‐haematopoietic transition (EHT).^1^ Elucidating the mechanisms of regulating embryonic haematopoiesis would allow us to establish an efficient strategy to generate functional haematopoietic cells in vitro. Human pluripotent stem cells (hPSCs), including human embryonic stem cells (hESCs) and human‐induced pluripotent stem cells (hiPSCs), bypass the limited access to primary human tissues to provide an easy approach to access to initial steps of haematopoiesis during human ontogeny and share a powerful platform for exploring the molecular dynamics that lead to human haematopoiesis.^2^, ^3^ Similar to embryonic haematopoietic development in vivo, haematopoietic differentiation of hPSCs in vitro can be generally divided into steps containing brachyury(T)^+^ mesodermal induction, CD34^+^CD144^+^ haemogenic endothelium (HE) specification and finally EHT for CD43^+^ haematopoietic progenitor cell (HPC) generation.^4^, ^5^, ^6^

The formation of mesoderm, as well as subsequent development of endothelial‐haematopoietic lineage, is highly organized and spatiotemporally controlled via the interaction of many cellular niches and other microenvironmental cues.^7^, ^8^ The extracellular matrix (ECM) plays a vital role in cell adhesion, migration, growth and differentiation and provides physical support for haematopoietic development, such as haematopoietic stem cell (HSC) proliferation and survival during homeostatic and stress conditions, as well as regulation of angiogenesis.^9^, ^10^, ^11^, ^12^ Deregulation of the balance between matrix deposition, degradation and cross‐linking results in haematopoietic impairment.^12^, ^13^ A better understanding of the role of ECM in contributing to haematopoietic development should lead to a novel option for further improving the efficiency of hPSC‐derived haematopoietic differentiation in vitro. The ECM contains various matrix proteins, such as collagens, fibronectin and vitronectin (VTN).^9^, ^10^ Of the different ECM proteins, collagens and fibronectin have been reported playing a role in HSC development and megakaryopoiesis.^14^, ^15^, ^16^, ^17^, ^18^ The collagens and fibronectin have also been used to promote haematopoietic and neural differentiation of hPSCs.^19^, ^20^, ^21^

VTN, as one of ECM members, is a multifunctional adhesive glycoprotein that directs cell adhesion and differentiation in many biological and pathological processes.^22^, ^23^ The αvβ3 and αvβ5 integrins, binding to the arginine‐glycine‐aspartate (RGD) domain of VTN to activate downstream signal transduction, are the key receptors to facilitate VTN function.^22^, ^23^ For example, VTN contributes to mouse angiogenesis by activating vascular endothelial growth factor receptor 2 (VEGFR‐2) via αvβ3.^24^ Similar to Matrigel (MTG), VTN is routinely used to replace mouse embryonic fibroblasts (MEFs) as feeder cells for hPSC maintenance and expansion.^25^, ^26^, ^27^ The effect of VTN on hPSC differentiation into haematopoietic cells has not been studied.

In the present work, we investigate the role of VTN in early haematopoietic differentiation of hPSCs and show that VTN promotes the development of haematopoietic‐fated mesoderm and further HE generation, compared with MTG. The αvβ3 and αvβ5 integrins were required for the promoting effect of VTN on early haematopoiesis. Inhibition of αvβ3 and αvβ5 signalling by specific inhibitors, such as Cilengitide or SB‐273005, impaired the HE development without affecting EHT. Our study of knockdown the expression of integrin genes of αvβ3 and αvβ5 confirmed that synergistic integrin signalling of αvβ3 and αvβ5 was necessary for HE development but not for EHT. Together, these findings establish a novel regulation mechanism of human early haematopoiesis and will shed light on the strategies for the production of clinically useful haematopoietic cells from hPSCs.

## 2 MATERIALS AND METHODS

### 2.1 Maintenance and differentiation of hPSCs

The H1 hESCs and hiPSC lines, including BC1, BC1‐T‐GFP, AD19B, 342‐3, 849‐3 and 342‐1, were grown on MTG (Corning)‐coated plates in E8 medium (Life Technologies), as described previously.^6^, ^8^, ^20^, ^28^, ^29^ For hPSC differentiation, single‐cell suspensions of hPSCs were obtained by treating the hPSC cultures at 70%‐80% confluency with TrypLE (Thermo Fisher Scientific). Single cells were then plated at an optimized density at 6 × 10^3^ cells/well onto 12‐well plates coated with different matrix or basement membrane proteins including VTN (PeproTech), MTG (Corning), growth factor‐reduced MTG (Corning), fibronectin (Corning), Tenascin C (Millipore) or Collagen IV (Sigma) in STEMdiff APEL 2 Medium (STEMCELL Technologies) supplemented with 3 μmol/L GSK3 inhibitor, CHIR99021 (abm Inc), 2 ng/mL ActivinA (PeproTech), 10 ng/mL BMP4 (PeproTech) and 10 µmol/L Rho kinase inhibitor, Y‐27632 (STEMCELL Technologies) on day 0. After 48 hours (day 2), the medium was changed to STEMdiff APEL 2 Medium supplemented with 40 ng/mL VEGF (PeproTech). For the following 24 hours (day 3), bFGF (abm Inc) was added to a final concentration of 20 ng/mL. From day 4, the medium was changed to STEMdiff APEL 2 Medium supplemented with 40 ng/mL VEGF and 20 ng/mL bFGF until day 6. From day 6, the medium was changed to STEMdiff APEL 2 Medium supplemented with 40 ng/mL VEGF, 20 ng/mL bFGF, 50 ng/mL SCF, 50 ng/mL TPO, 50 ng/mL FLT3L, 20 ng/mL IL‐3 and 20 ng/mL IL‐6 until day 10. Specific shRNAs against *ITGAV*, *ITGB3* or *ITGB5* were designed and chemically synthesized by OBiO Co. and used as indicated. The entire differentiation process was incubated at 37°C in 5% CO_2_ with 100% humidity. Where indicated, Cilengitide (500 nmol/L, Selleck), SB‐273005 (10 nmol/L, Selleck) and ATN‐161 (10 µmol/L, MCE) were included.

### 2.2 Endothelial‐to‐haematopoietic transition (EHT) assay

CD34^+^CD144^+^CD43^−^CD73^−^CD184^−^ cells were isolated from differentiated cells on day 4 by FACSAria III cell sorter (BD Biosciences). For EHT culture, the isolated CD34^+^CD144^+^CD43^−^CD73^−^CD184^−^ cells were re‐seeded on VTN‐coated plates for an additional 4 days in STEMdiff APEL 2 Medium supplemented with SCF (100 ng/mL, PeproTech), TPO (100 ng/mL, PeproTech), FLT3‐L (100 ng/mL, PeproTech), IL‐3 (20 ng/mL, PeproTech), IL‐6 (20 ng/mL, PeproTech), VEGF (40 ng/mL, PeproTech) and bFGF (20 ng/mL, abm Inc). Cultures were incubated at 37°C in 5% CO_2_ with 100% humidity. After 4 days of EHT culture, the cells were collected by TrypLE for further analysis.

### 2.3 Flow cytometry analysis

Cells were dissociated to form a single‐cell suspension by TrypLE treatment and washed with FACS buffer (1% FBS and 1 mmol/L EDTA in PBS). The dissociated cells were resuspended in FACS buffer and labelled with fluorochrome‐conjugated anti‐human CD34‐APC/Cyanine7 (clone 561, BioLegend), KDR‐PE (clone ES8‐20E6, Miltenyi Biotec), CD31‐FITC (clone AC128, Miltenyi Biotec), CD144‐APC (clone 16B1, eBioscience), CD43‐PerCP (clone TP1/36, Abcam), CD45‐APC (clone 2D1, BioLegend), CD144‐PE (clone BV9, BioLegend), CD43‐FITC (clone MEM‐59, BioLegend), CD73‐PE/Cyanine7 (clone AD2, BioLegend), CD184‐APC (clone 12G5, BioLegend), CD51/61‐FITC (clone 23C6, BioLegend), integrin β5‐PE (clone AST‐3T, BioLegend) and APLNR‐Alexa Fluor 647 (clone 72133R, RD system). Dead cells were excluded by DAPI (BD Biosciences) staining. Isotype‐matched control antibodies were used to determine the background staining. Flow cytometry was performed on LSR II or Canto II analyser (BD Biosciences). Data analysis was performed using FlowJo software (Tree Star, Inc).

### 2.4 Haematopoietic colony‐forming cell (CFC) assays

Single cells of the indicated numbers in 0.1 mL IMDM (Life Technologies) with 2% FBS were mixed with 1 mL MethoCult H4034 Optimum (STEMCELL Technologies). The mixture was then transferred to 2 wells of ultra‐low attachment 24‐well plates (Corning). The cells were incubated at 37°C in 5% CO_2_ with 100% humidity for 14 days, and then, the colonies were counted. Each type of colony was classified according to morphology. Each assay was performed in triplicate.

### 2.5 RNA‐sequencing

The day 6 VTN or MTG‐coated cells were collected for RNA‐sequencing (RNA‐seq). The RNA‐seq library construction, sequencing and analysis were performed by NovoGene. Differential expression analysis was performed using the DESeq2 R package (1.16.1). Gene Ontology (GO) enrichment analysis of differentially expressed genes was implemented by the clusterProfiler R package. The results are available at Sequence Read Archive with the accession number of PRJNA692000.

### 2.6 Quantitative real‐time polymerase chain reaction (qRT‐PCR) assay

Total RNA was extracted from cells using a RNeasy Mini Kit (Qiagen) and treated with RNase‐free DNase (Qiagen). cDNAs were synthesized with random hexamers and Oligo(dT) with Superscript III Reverse Transcriptase (Invitrogen) and stored at −20°C until use. Real‐time PCR was performed using a FastStart Universal SYBR Green Master (Roche) on a QuantStudio™ 3 (Life Technologies). Amplification of β‐actin was also conducted to control the quantity of loaded cDNA in each reaction. Primers sequences are listed in Table S1.

### 2.7 Statistical analysis

Data obtained from multiple experiments were reported as the mean ± SEM. An unpaired *t* test was used to compare the means from two groups, and ANOVA was used to compare the means from three groups or more. Results with a value of *P* < .05 were considered statistically significant. *P < .05; **P < .01; ***P < .001.

## 3 RESULTS

### 3.1 Vitronectin promotes early haematopoiesis of hPSCs

**FIGURE 1:**
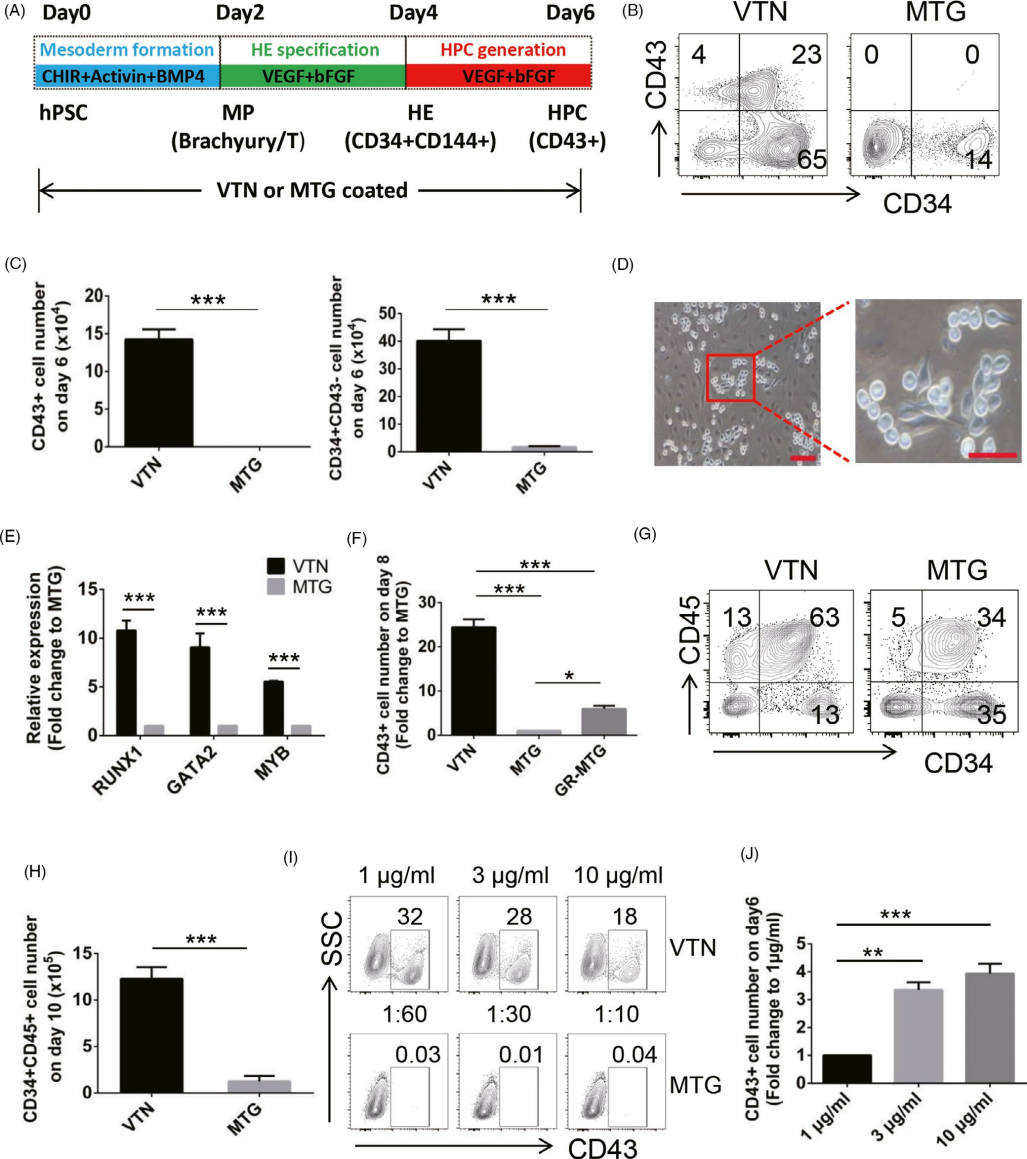
VTN promotes early haematopoietic differentiation of hPSCs, compared to MTG. A, Schematic showing the strategy for the generation of hPSC‐derived haematopoietic cells in VTN or MTG‐coated cultures. B and C, Flow cytometric analysis of the frequency and number of CD43^+^ and CD34^+^CD43^‐^ cells on day 6. n = 3. D, The representative images showed that haematopoietic‐like cells with round shape and endothelium‐like cells undergoing EHT emerged in VTN‐coated culture on day 6. Scale bars, left 100 µm, right 50 µm. n = 3. E, qRT‐PCR analysis of *RUNX1*, *GATA2* and *MYB* expression in the day 6 cells coated with VTN or MTG. n = 3. F, CD43^+^ cell number on day 8 generated in VTN, MTG or GR‐MTG‐coated culture. MTG was set as a control and normalized to 1. n = 3. G and H, Flow cytometric analysis of the frequency and number of CD34^+^ CD45^+^ cells on day 10 generated in VTN or MTG‐coated culture. n = 6. I, Representative flow cytometric analysis of the CD43 expression in the day 6 cells coated with different concentrations of VTN or MTG. n = 3. J, The cell number of CD43^+^ cells generated in different concentrations of VTN on day 6. 1 μg/mL was set as a control and normalized to 1. n = 3. Experiments were performed on H1 unless otherwise indicated

VTN and MTG are routinely used for hPSC culture.^26^, ^27^, ^30^ To investigate their roles in haematopoiesis, we compared the effect of VTN and MTG on haematopoietic differentiation of hPSCs by using a stepwise monolayer differentiation system adapted from our previously established protocol.^6^ The development of Brachyury^+^ mesodermal progenitors (MPs) was facilitated by Activin A, BMP4 and Wnt signalling activator (CHIR99021), and the development of CD34^+^CD144^+^ HE cells and CD43^+^ HPCs was induced by VEGF and bFGF (Figure 1A). As shown in Figure 1B,C, VTN effectively promoted the generation of CD43^+^ HPCs and CD34^+^CD43^−^ endothelial cells assessed by flow cytometry on day 6. Compared with VTN and MTG, the mesoderm‐like cells on day 2 were similar morphologically; however, VTN effectively promoted the differentiation and proliferation of endothelial like cells on day 4 and day 6 (Figure S1A). On day 6, haematopoietic‐like cells with round shape and endothelium‐like cells undergoing EHT were clearly observed in VTN‐coated culture (Figure 1D) and the typical budding process of EHT on VTN was further captured by timelapse imaging revealing that the slender endothelium‐like cells gradually acquired round shape with CD43 expression (in red) and then gave rise to two semi‐adherent round shape CD43+ haematopoietic cells (Video S1). We then examined the gene expression profile of key haematopoietic transcription factors, including *RUNX1*, *GATA2* and *MYB*, in the differentiated day 6 cells cultured on VTN or MTG. Indeed, the expression of *RUNX1*, *GATA2* and *MYB* in VTN‐coated culture was significantly higher than that in MTG‐coated culture (Figure 1E). To further determine the haematopoietic potential of VTN or MTG‐coated culture, we extended the culture to assess the generation of CD43+ HPCs on day 8 and day 10 and CD34^+^CD45^+^ haematopoietic stem and progenitor cells (HSPCs) on day 10 by adding both haematopoietic and endothelial growth factors from day 6. Compared with MTG‐coated culture, VTN effectively promoted the generation of CD43^+^ HPCs on day 8 and day 10 (Figure 1F and Figure S1B,C) and CD34^+^CD45^+^ HSPCs on day 10 (Figure 1G,H). A more defined MTG, growth factor‐reduced Matrigel (GR‐MTG), was tested for haematopoietic differentiation, compared with VTN and MTG. As shown in Figure 1F, GR‐MTG modestly enhanced CD43+ HPC generation, suggesting that MTG contains inhibitory factors for haematopoietic differentiation. We then assessed the effect of different concentrations of VTN or MTG on CD43^+^ HPC generation. Although the frequency of CD43^+^ cells was increased in a low VTN concentration (1 µg/mL), the number of CD43^+^ cells was decreased in a low VTN concentration (Figure 1I,J). However, altering MTG concentration had no effect on the generation of CD43^+^ HPCs in MTG‐coated culture (Figure 1I). Considering the coating with 3 µg/mL VTN or 10 µg/mL VTN shows no significant effect on the production of CD43^+^ HPCs (Figure 1J), 3 µg/mL VTN was applied in our further study. Other EMCs, such as fibronectin, Tenascin C and Collagen IV, have been reported playing a role in haematopoietic development.^15^, ^18^, ^19^ We also compared their roles in haematopoietic differentiation of hPSCs with VTN. The total numbers of CD43^+^ HPCs from cultures with fibronectin, Tenascin C or Collagen IV were all decreased; however, fibronectin, Tenascin C or Collagen IV had similar frequency of CD43^+^ HPCs on day 6 (Figure S1D,E).

### 3.2 hPSC‐derived HPCs are enriched in semi‐attached cell clusters

**FIGURE 2:**
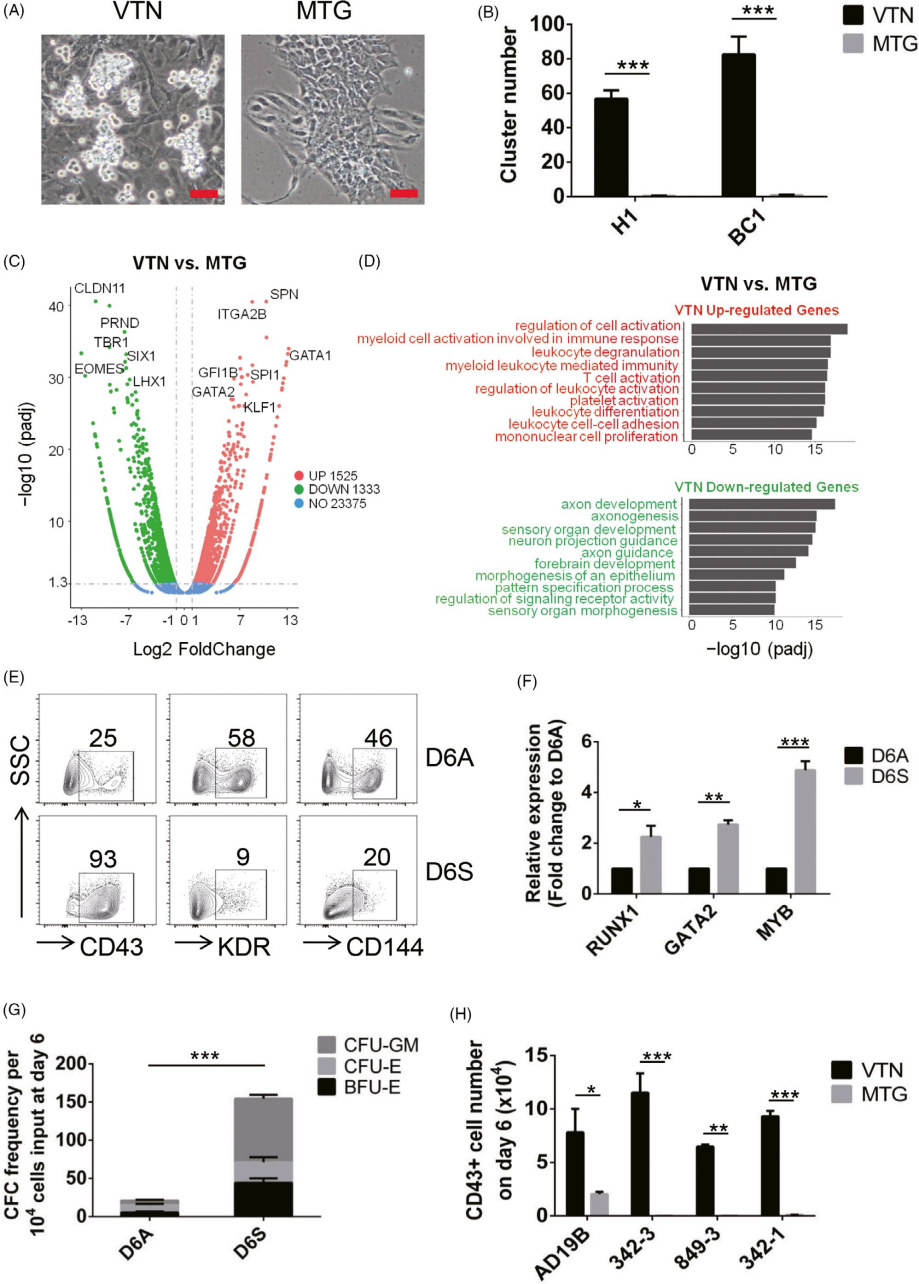
CD43^+^ HPCs are enriched in semi‐attached cell cluster. A, Photomicrograph of the day 6 cells coated with VTN or MTG. Semi‐adherent cell clusters (≥3 cells) are visible in the VTN cultures. Scale bars, 50 µm. B, The number of semi‐adherent cell clusters per visual unit in VTN or MTGl‐coated cultures on day 6. H1‐hESC and BC1‐hiPSC were applied. n = 3. C, Volcano plot displaying the differentially expressed genes (DEGs) in VTN or MTG‐coated cultures on day 6. Representative upregulated (red) and downregulated (green) genes are indicated. Blue dots represent non‐DEGs. D, GO analysis of downregulated and upregulated genes on day 6 comparing VTN with MTG. E, Representative flow cytometric analysis of the frequency of CD43^+^, KDR^+^ and CD144^+^ cells in the day 6 adherent or semi‐adherent cells. n = 3. F, qRT‐PCR analysis of *RUNX1*, *GATA2* and *MYB* expression in the day 6 adherent (D6A) or semi‐adherent cells (D6S). n = 3. G, Haematopoietic colony‐forming potential of the day 6 adherent (D6A) or semi‐adherent cells (D6S). CFUs per 10 000 cells plated. n = 3. BFU‐E, burst‐forming unit‐erythroid; CFU‐E, colony‐forming unit‐erythroid; CFU‐GM, colony‐forming unit‐granulocyte, macrophage. H, The number of AD19B, 342‐3, 849‐3 and 342‐1‐hiPSC‐derived CD43^+^ cells on day 6 in VTN or MTG‐coated cultures. n = 3. Experiments were performed on H1 unless otherwise indicated

We observed that there were significantly increased semi‐adherent cell clusters on VTN‐coated plates on day 6 either from H1 hESCs or BC1 hiPSCs, compared with MTG‐coated plates (Figure 2A,B). To compare the molecular changes between VTN and MTG, a transcriptome analysis was applied after 6 days of haematopoietic differentiation. The genes in VTN‐cultured cells were strongly enriched for the GO terms related to haematopoietic differentiation, including *SPN*, *GATA2*, *SPI1*, *GFI1B* and *KLF1*, and were significantly upregulated (Figure 2C,D and Figure S2A,B). Downregulated genes in VTN‐cultured cells were related to neurogenesis and were mainly enriched for axon development (Figure 2C,D and Figure S2A,B). To further investigate whether the semi‐adherent clusters possess haematopoietic potential, the semi‐adherent and adherent cells in VTN‐coated culture were separately harvested and analysed by flow cytometry. Most of the semi‐adherent cells (93%) were CD43^+^ HPCs with relatively low expression of endothelial markers of KDR and CD144, while the adherent cells highly expressed endothelial markers with low CD43 expression (Figure 2E). qRT‐PCR analysis showed that the expression of *MYB*, *RUNX1* and *GATA2* was significantly higher in semi‐adherent cells (D6S) than in adherent cells (D6A) (Figure 2F). We then used colony‐forming unit (CFU) assay to functionally assess the haematopoietic progenitor potential in semi‐adherent cells and in adherent cells. As shown in Figure 2G and Figure S2C, the semi‐adherent cells gave rise to a significant increased number of haematopoietic colonies, compared to the adherent cells. The VTN‐promoting effect on CD43^+^ HPC generation was also observed in another four hiPSC lines, including AD19B, 342‐3, 849‐3 and 342‐1, on day 6 and day 8 (Figure 2H and Figure S2D).

### 3.3 VTN promotes the specification of haematopoietic‐fated mesoderm and enhances HE generation from mesodermal progenitor cells

**FIGURE 3:**
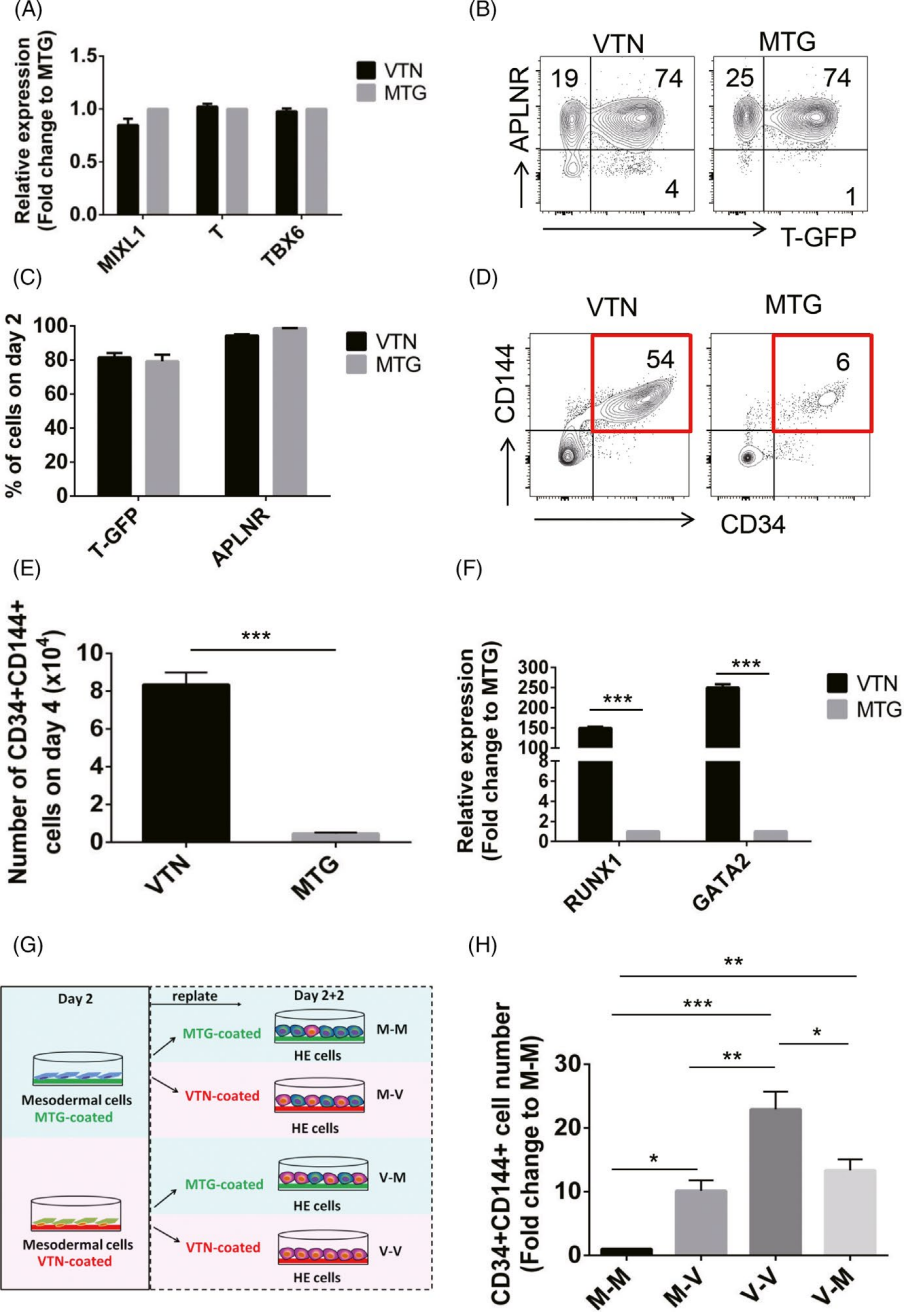
VTN specifies haematopoietic‐fated mesoderm and enhances HE generation from the mesoderm. A, qRT‐PCR analysis of *MIXL1*, *T* and *TBX6* expression in the day 2 VTN or MTG‐coated cells. n = 3. B and C, Flow cytometric analysis of the frequency of T‐GFP^+^ and APLNR^+^ cells in the day 2 cells coated with VTN or MTG. n = 3. D and E, Flow cytometric analysis of the frequency and number of CD34^+^CD144^+^ cells in the day 4 VTN or MTG‐coated cells. n = 3. F, qRT‐PCR analysis of *RUNX1* and *GATA2* expression in the day 4 cells coated with VTN or MTG. n = 3. G, Scheme depicting the strategy used for evaluating the role of VTN in the fate determination of mesoderm cells and their development into HE cells. The day 2 T‐GFP + mesodermal cells coated with VTN or MTG were sorted and re‐seeded on VTN and MTG, respectively, for an additional 2 days of HE induction. H, The fold change of CD34^+^CD144^+^ cell number generated in the day 2 T‐GFP + mesodermal cells coated with VTN or MTG following an additional two‐day culture in VTN or MTG. M‐M was set as a control and normalized to 1. n = 3. Experiments were performed on H1 unless otherwise indicated

To understand how VTN promotes haematopoietic development, we then examined the VTN‐dependent haematopoietic differentiation of hPSCs at different developmental stages. We first analysed the expression of key genes associated with mesoderm development, including *MIXL1*, *Brachyury (T)* and *TBX6,* by qRT‐PCR on day 2 in H1 hESCs. There were no significant differences in mesodermal‐specific gene expression in cells cultured with VTN or MTG (Figure 3A). We then tracked the development of mesoderm using a Brachyury (T)‐GFP reporter system (T‐GFP) in BC1 hiPSCs, as we previously described.^6^ Flow cytometry analysis of T‐GFP and APLNR (apelin receptor) expressing cells on day 2 indicated that there was no difference of T‐GFP^+^APLNR^+^ mesodermal progenitors between VTN‐ and MTG‐coated cultures (Figure 3B,C). By tracking the total cell numbers between VTN‐ and MTG‐coated cultures, we found that VTN‐ and MTG‐coated cultures gave a similar number of cells on day 2; however, VTN‐coated cultures significantly increased the total number of cells on day 3 and day 4 (Figure S3A).

To examine whether VTN promotes HE development, we analysed CD34^+^CD144^+^ cells by flow cytometry on day 4. Compared with MTG‐coated culture, VTN significantly increased the frequency and number of CD34^+^CD144^+^ cells (Figure 3D,E). Most of the CD34^+^CD144^+^ cells co‐expressed CD31 and KDR (Figure S3B). qRT‐PCR analysis on day 4 showed that the key genes associated with HE, including *RUNX1* and *GATA2,* were significantly higher in cells cultured in VTN than in MTG (Figure 3F).

To further determine the developmental potential of mesodermal progenitors, we assessed the effect of VTN on the development of haematopoietic‐fated mesodermal progenitors. The T‐GFP^+^ mesodermal progenitors generated in VTN‐ or MTG‐coated cultures on day 2 were isolated and then re‐seeded on either VTN‐ or MTG‐coated plates for additional 2 days, respectively. The CD34^+^CD144^+^ cells were analysed by flow cytometry on day 4 (day 2 + 2) (Figure 3G). When mesodermal progenitors from VTN or MTG‐coated culture were re‐plated on MTG (V‐M or M‐M), we found that the VTN‐primed mesodermal progenitors possessed a higher potential to differentiate into CD34^+^CD144^+^ cells (Figure 3H). When the mesodermal progenitors generated in VTN‐ or MTG‐coated culture were re‐plated on VTN (V‐V or M‐V), VTN‐primed mesodermal progenitors also gave rise to an increased number of CD34^+^CD144^+^ cells (Figure 3H). When MTG‐coated mesoderm cells were re‐seeded on MTG or VTN respectively (M‐M or M‐V), VTN significantly promoted the differentiation of mesodermal cells into CD34^+^CD144^+^ cells (Figure 3H). Similar results were found when VTN‐coated mesodermal cells were re‐seeded on MTG or VTN (V‐M, V‐V) (Figure 3H). Collectively, these data suggest that (a) VTN promotes the development of mesodermal progenitors with haematopoietic fate, and (b) VTN promotes HE development from mesodermal cells.

### 3.4 VTN promotes early haematopoiesis of hPSCs via αvβ3 and αvβ5 integrins

**FIGURE 4:**
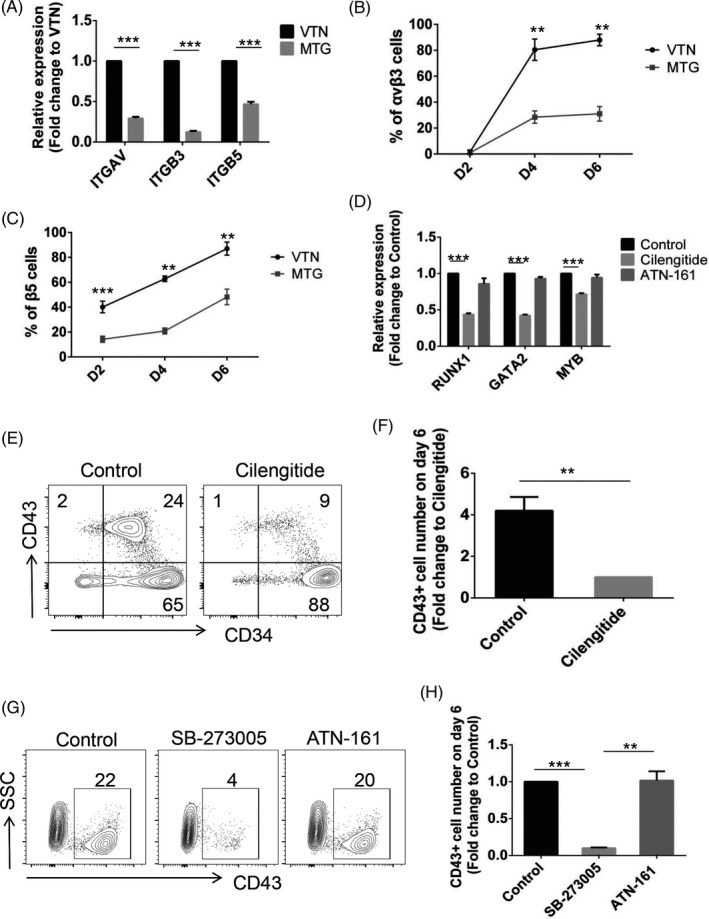
VTN promotes early haematopoietic differentiation of hPSCs via αvβ3 and αvβ5 integrins. A, qRT‐PCR analysis of *ITGAV*, *ITGB3* and *ITGB5* expression in the day 2 VTN or MTG‐coated cells. VTN was set as a control and normalized to 1. n = 3. B and C, Flow cytometry analysis of the expression of αvβ3 and β5 integrins on day 2, day 4 and day 6 in VTN or MTG‐coated cultures. n = 3. D, qRT‐PCR analysis of *RUNX1*, *GATA2* and *MYB* expression in the day 6 VTN‐coated cells treated with or without Cilengitide or ATN‐161 from day 2 to day 6. Control was normalized to 1. n = 3. E and F, Flow cytometric analysis of the frequency and number (fold change) of CD43^+^ cells in the day 6 VTN‐coated cells treated with or without Cilengitide from day 2 to day 6. Cilengitide was set as a control and normalized to 1. n = 3. G and H, Flow cytometric analysis of the frequency and number (fold change) of CD43^+^ cells in the day 6 VTN‐coated cells treated with or without SB‐273005 or ATN‐161 from day 2 to day 6. Control was normalized to 1. n = 3. Experiments were performed on H1 unless otherwise indicated

Considering that αvβ3 and αvβ5 integrins mediated VTN function in angiogenesis,^24^, ^31^, ^32^ we tested whether signalling of αvβ3 and αvβ5 integrins was required for VTN‐promoted haematopoietic development. Despite the fact that the mesodermal progenitors derived from VTN or MTG expressed similar mesodermal markers of *MIXL1*, *Brachyury (T)* and *TBX6* (Figure 3A), the gene expression levels of integrin genes *ITGAV*, *ITGB3* and *ITGB5* were significantly higher in VTN‐coated mesodermal progenitors than that in MTG (Figure 4A), suggesting that VTN altered intrinsic signals in emerged mesodermal progenitors. We also performed flow cytometry to dynamically track the expression of αvβ3 and β5 integrins on day 2, day 4 and day 6. The cells positive for αvβ3 and β5 were significantly increased on VTN, compared to MTG (Figure 4B,C and Figure S4).

To examine whether signalling through αvβ3 and αvβ5 integrins was required for haematopoietic development, we used a small molecular inhibitor, Cilengitide that is the integrin antagonist of αvβ3 and αvβ5 integrins, to block integrin signalling during hPSC differentiation in VTN‐coated culture. An addition of Cilengitide during the first two days of differentiation seriously impaired cell adhesion (Figure S5A,B). Nuclear staining indicated that cell survival was not affected by Cilengitide (Figure S5C‐E). To examine whether the signalling of αvβ3 and αvβ5 integrins is required for the haematopoietic development of mesodermal progenitor, Cilengitide was added on day 2 after cell attachment for mesodermal development in VTN‐coated culture. After 4 days of Cilengitide treatment, a decreased expression of key genes related to haematopoiesis, including *RUNX1*, *GATA2* and *MYB*, was observed (Figure 4D). The addition of an antagonist of integrin α5β1, ATN‐161, had no effect on the expression of *RUNX1*, *GATA2* and *MYB* (Figure 4D). Flow cytometry analysis indicated that Cilengitide treatment from days 2 to 6 significantly decreased the frequency and the number of CD43^+^ HPCs, compared with control group (Figure 4E,F). The requirement of αvβ3 and αvβ5 signalling for haematopoietic development was confirmed by another αvβ3 and αvβ5 inhibitor, SB‐273005, while ATN‐161 do not affect the production of CD43^+^ HPCs (Figure 4G,H).

### 3.5 αvβ3 and αvβ5 inhibition impairs HE development without affecting EHT

**FIGURE 5:**
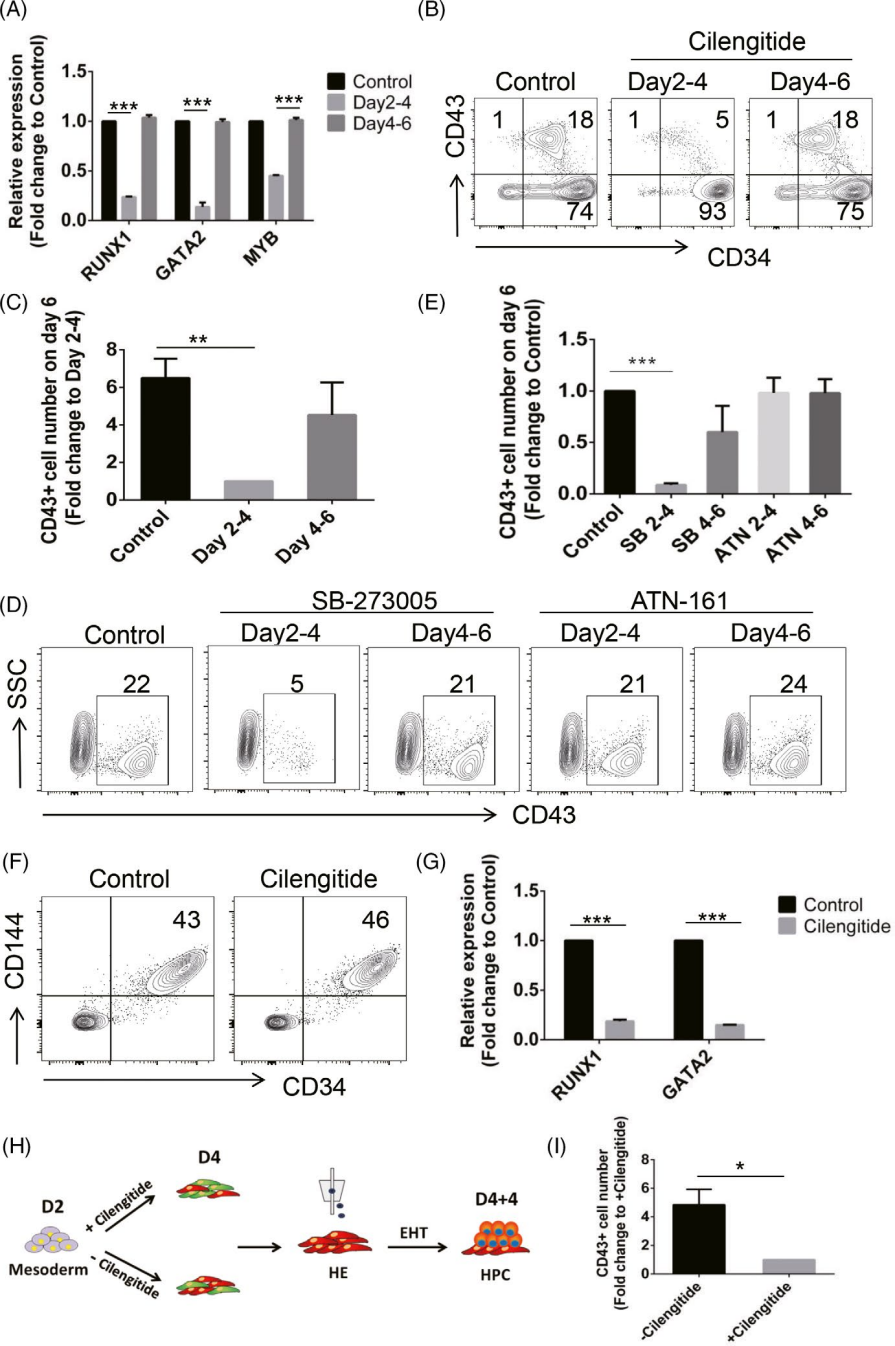
Inhibition of αvβ3 and αvβ5 integrins impairs HE development without affecting EHT. A, qRT‐PCR analysis of *RUNX1*, *GATA2* and *MYB* expression in the day 6 VTN‐coated cells treated with or without Cilengitide at Days 2‐4 or Days 4‐6. Control was normalized to 1. n = 3. B and C, Flow cytometric analysis of the frequency and number (fold change) of CD43^+^ cells in the day 6 VTN‐coated cells treated with or without Cilengitide at Days 2‐4 or Days 4‐6. Control was normalized to 1. n = 3. D and E, Flow cytometric analysis of the frequency and number (fold change) of CD43^+^ cells in the day 6 VTN‐coated cells treated with or without SB‐273005 or ATN‐161 at Days 2‐4 or Days 4‐6. Control was normalized to 1. n = 3. F, Representative flow cytometric analysis of the frequency of day 4 CD34^+^CD144^+^ cells treated with or without Cilengitide at Days 2‐4. n = 3. G, qRT‐PCR analysis of *RUNX1* and *GATA2* expression in the day 4 cells treated with or without Cilengitide at Days 2‐4. Control was normalized to 1. n = 3. H, Scheme depicting the strategy used for evaluating the haematopoietic potential of day 4 CD34^+^CD144^+^CD43^−^CD73^−^CD184^−^ cells treated with or without Cilengitide at Days 2‐4. The day 4 CD34^+^CD144^+^CD43^−^CD73^−^CD184^−^ cells treated with or without Cilengitide at Days 2‐4 were sorted and then re‐seeded on VTN‐coated plates for an additional 4 days of EHT culture for HPC generation. I, The number of CD43^+^ cells generated from the day 4 CD34^+^CD144^+^CD43^−^CD73^−^CD184^−^ cells treated with or without Cilengitide at Days 2‐4 following an additional 4 days of EHT culture. n = 3. Experiments were performed on H1 unless otherwise indicated

To examine the involvement of αvβ3 and αvβ5 integrin signalling in HE development and EHT, we added integrin inhibitors at different time points for a 2‐day interval from day 2 to day 4 (Days 2‐4) for HE development and day 4 to day 6 (Days 4‐6) for EHT, respectively. The expression of haematopoietic genes (*RUNX1*, *GATA2* and *MYB)* and the generation of CD43^+^ HPCs were impaired by Cilengitide treatment at Days 2‐4, but not at Days 4‐6 (Figure 5A‐C), suggesting that αvβ3 and αvβ5 inhibition impaired HE development, but not EHT. Similar to Cilengitide treatment, SB‐273005 treatment at Days 2‐4, but not at Days 4‐6, significantly decreased the frequency and the number of CD43^+^ HPCs (Figure 5D,E). As expected, the α5β1 antagonist of ATN‐161 neither impaired the gene expression of haematopoietic markers (*RUNX1*, *GATA2* and *MYB*) (Figure S6), nor the production of CD43+ HPCs (Figure 5D,E).

To determine whether αvβ3 and αvβ5 integrin signalling is required for endothelial development or specific for HE development, we treated cells with Cilengitide for 2 days between day 2 and day 4, and analysed CD34 and CD144 by flow cytometry on day 4. To our surprise, the frequency of CD34^+^CD144^+^ cells was not altered by Cilengitide treatment (Figure 5F). Further analysis showed that the CD34^+^CD144^+^ cells treated with or without Cilengitide expressed little CD43, CD73 or CD184 (Figure S7A). Interestingly, the expression of key genes associated with HE cells, including *RUNX1* and *GATA2*, was decreased by Cilengitide treatment (Figure 5G), suggesting inhibition of αvβ3 and αvβ5 signalling at Days 2‐4 impaired CD34^+^CD144^+^ cells to acquire haematopoietic potential. To confirm the role of αvβ3 and αvβ5 integrin signalling in HE generation, CD34^+^CD144^+^CD43^−^CD73^−^CD184^−^ cells treated with or without Cilengitide for 2 days (Days 2‐4) were sorted on day 4 and then re‐seeded for additional 4 days (D4 + 4) of EHT culture (Figure 5H). Indeed, the haematopoietic potential to generate CD43^+^ HPCs was impaired in CD34^+^CD144^+^CD43^−^CD73^−^CD184^−^ cells pre‐treated with Cilengitide (Figure 5I). To further determine the effect of αvβ3 and αvβ5 integrin signalling on EHT, the day 4 CD34^+^CD144^+^CD43^−^CD73^−^CD184^−^ cells were sorted and then re‐seeded for additional 4 days (D4 + 4) of EHT culture with or without Cilengitide treatment (Figure S7B). As shown in Figure S7C,D, the treatment with Cilengitide during EHT did not affect the HE cells to generate CD43 + HPCs. Taken together, these data demonstrate that VTN signalling through αvβ3 and αvβ5 integrins is essential for the development of HE to acquire haematopoietic potential, but not EHT.

### 3.6 Both αvβ3 integrin signalling and αvβ5 integrin signalling are required for HE development, but not for EHT

**FIGURE 6:**
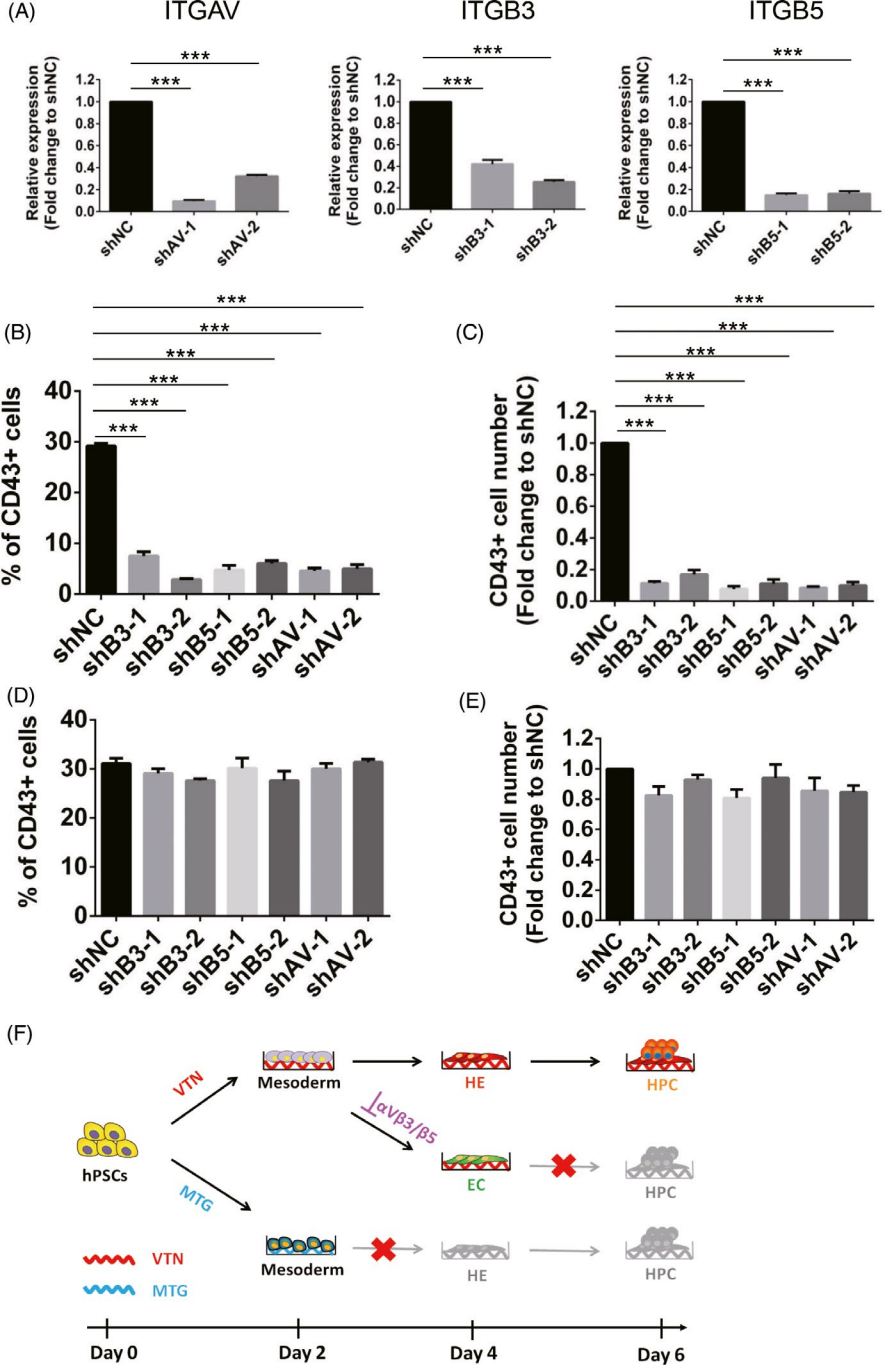
Both αvβ3 integrin signalling and αvβ5 integrin signalling are required for HE development, but not for EHT. A, qRT‐PCR analysis of *ITGAV*, *ITGB3* and *ITGB5* expression in the day 6 VTN‐coated cells treated with specific shRNA against *ITGAV* (shAV‐1 and shAV‐2), *ITGB3* (shB3‐1 and shB3‐2) or *ITGB5* (shB5‐1 and shB5‐2) at Days 4‐6. Negative control shRNA (shNC) was set as a control and normalized to 1. n = 3. B and C, Flow cytometric analysis of the frequency and number (fold change) of CD43^+^ cells in the day 6 VTN‐coated cells treated with specific shRNA against *ITGAV* (shAV‐1 and shAV‐2), *ITGB3* (shB3‐1 and shB3‐2) or *ITGB5* (shB5‐1 and shB5‐2) at Days 2‐4. Negative control shRNA (shNC) was set as a control and normalized to 1. n = 3. D and E, Flow cytometric analysis of the frequency and number (fold change) of CD43^+^ cells in the day 6 VTN‐coated cells treated with specific shRNA against *ITGAV* (shAV‐1 and shAV‐2), *ITGB3* (shB3‐1 and shB3‐2) or *ITGB5* (shB5‐1 and shB5‐2) at Days 4‐6. Negative control shRNA (shNC) was set as a control and normalized to 1. n = 3. F, Model of VTN‐mediated haematopoietic differentiation from hPSCs. Compared with MTG, VTN was required for the mesoderm to acquire higher endothelial‐haematopoietic potential. The promoting effect of VTN on early haematopoiesis was dependent on αvβ3 and αvβ5 integrins. Inhibition of αvβ3 and αvβ5 impaired HE development but without affecting EHT. Experiments were performed on H1 unless otherwise indicated

To further determine whether αvβ3 or αvβ5 or both are required for HE development, we used shRNAs to silence the integrin expression. Specific shRNAs against *ITGAV* (shAV‐1 and shAV‐2), *ITGB3* (shB3‐1 and shB3‐2) and *ITGB5* (shB5‐1 and shB5‐2) were employed to knockdown the expression of *ITGAV*, *ITGB3* and *ITGB5*, respectively. As shown in Figure 6A, transfection of shRNAs into differentiated cells caused significant decreases in *ITGAV*, *ITGB3* and *ITGB5* mRNA levels, while a negative control shRNA (shNC) had no effect on the expression of *ITGAV*, *ITGB3* and *ITGB5* as well as CD43+ HPCs (Figure S8A‐C), suggesting the high effectiveness of our synthesized shRNAs. When shRNAs were applied to the cells at the stage of HE development (Days 2‐4), the frequency and the number of CD43+ HPCs were significantly decreased by knockdown of either *ITGB3*, *ITGB5* or *ITGAV* (Figure 6B,C). To determine the effect of specific integrins on EHT, we added shRNAs from days 4 to 6, and collected cells for flow cytometry on day 6. We found that there was no effect on the proportion and production of CD43+ HPCs by knockdown of either *ITGB3*, *ITGB5* or *ITGAV* (Figure 6D,E). Taken together, these data demonstrate that both αvβ3 integrin signalling and αvβ5 integrin signalling are required for HE development, but not for EHT (Figure 6F).

## 4 DISCUSSION

The roles of ECM and microenvironment in regulating lineage development from hPSCs remain to be elucidated. VTN is one of ECM proteins, which has been commonly used for hPSC expansion as the feeder‐free culture system. Here, we focused on the role of VTN in regulating haematopoietic differentiation of hPSCs. The main findings of our study are that (a) VTN promotes early haematopoiesis of hPSCs by specification of haematopoietic‐fated mesoderm, and enhances HE generation from mesodermal progenitors, (b) the effects of VTN on haematopoietic development are mediated by αvβ3 and αvβ5 integrins, and (c) αvβ3 and αvβ5 inhibition impairs HE development without affecting EHT. These results demonstrate that VTN plays an important role in human haematopoietic development.

Since the first study of haematopoietic differentiation from human embryonic stem cells,^33^ numerous studies have been conducted and led to successful derivation of a broad spectrum of blood cell lineages from hPSCs through co‐culture with stromal cells or the formation of embryoid bodies (EBs).^34^, ^35^, ^36^, ^37^ However, the use of xenogeneic or allogeneic feeder cells, and poorly defined serum limits the utility of the current differentiation systems for studying factors that are essential for haematopoietic development and specification, whereas the sphere‐like structure of EBs slows the penetration of cytokines and other microenvironment signals into EBs thus delays their effects. Here, we show that VTN, a chemically defined ECM protein, can be applied to promote haematopoietic differentiation of hPSCs. Our study of VTN‐based serum‐free haematopoietic system would present a novel platform for further improving the efficiency to investigate the molecular programmes involved in human haematopoietic development.

Previous studies have shown that different ECM proteins play distinct roles in haematopoiesis.^16^, ^18^, ^38^, ^39^ To objectively evaluate the effect of VTN on early haematopoiesis, we applied different matrix or basement membrane proteins, including VTN, fibronectin, Tenascin C, Collagen IV and MTG, in our monolayer‐based haematopoietic differentiation system. Consistent with previous reports, either fibronectin, Tenascin C or Collagen IV showed haematopoietic supporting potential to generate CD43^+^ HPCs on day 6 (Figure S1D,E). Compared to VTN, fibronectin, as well as Tenascin C and Collagen IV, showed similar frequency but decreased number of CD43^+^ HPCs (Figure S1D,E). By comparing the role of VTN with MTG, it is surprising that there are few CD43+ HPCs generated on day 6 (Figure 1B,C). When extended the culture to days 8 and 10, there are still few CD43+ HPC generation in MTG (Figure 1F and Figure S1B,C), suggesting that VTN is favoured for haematopoietic differentiation of hPSCs. Since MTG is a complex mix of basement membrane proteins containing growth factors and cytokines, there are many possible components that could inhibit haematopoietic differentiation. This notion was supported by using a growth factor‐reduced MTG (GR‐MTG), which enhanced CD43+ HPC generation, compared to MTG (Figure 1F and Figure S1B).

Within the matrix, VTN supports cellular adhesion via interactions with many integrins, including αvβ1, αvβ3, αvβ5, αvβ6, αvβ8, α8β1 and αIIbβ3.^40^, ^41^, ^42^, ^43^ During early haematopoietic differentiation of hPSCs, we found that αvβ3 and αvβ5 were crucial for cell adhesion for mesodermal development, the generation of haematopoietic‐fated mesodermal progenitors, and the HE generation, consistent with a previous study showing that the αvβ5 was required for initial attachment of hiPSCs on VTN, but inhibition of both αvβ5 and β1 was required to significantly decrease hiPSC proliferation.^44^ Although we cannot exclude a possible involvement of multiple VTN‐binding integrins, it is likely that VTN‐promoted HE specification is mediated mostly through integrins αvβ3 and αvβ5, as both are reported as high expressing on endothelial cells.^23^, ^45^ The integrins αvβ3 and αvβ5 are found to be crucial for endothelial cell survival,^32^, ^46^ and the survival signals transmitted by integrin αvβ3 lead to inhibition of p53 activity, decreased expression of p21WAF1/CIP1 and suppression of the Bax cell death pathway in endothelial cells.^47^ NFκB has also been identified as an important signalling molecule in αvβ3 integrin‐mediated endothelial cell survival.^48^ Compared to these studies, although we showed that the inhibition of αvβ3 and αvβ5 impaired mesodermal adhesion and HE generation, the downstream signalling pathways of these integrins are still not understood.

## CONFLICTS OF INTEREST

No competing financial interests exist.

## AUTHOR CONTRIBUTIONS

JS, YZ and SZ involved in conception and design, collection and assembly of data, data analysis and interpretation, manuscript writing and final approval of manuscript; CL, ZF and SL involved in collection and assembly of data, data analysis and interpretation, and final approval of manuscript; DLH wrote the manuscript and involved in final approval of manuscript; ZZW and TC involved in conception and design, financial support, administrative support, collection and assembly of data, data analysis and interpretation, manuscript writing and final approval of manuscript.

## Supporting information

Supplementary Material

Video S1

## Data Availability

The data that support the findings of this study are available on request from the corresponding author.
