# Supplementary material for: Vitronectin‐activated αvβ3 and αvβ5 integrin signalling specifies haematopoietic fate in human pluripotent stem cells
Source: Cell Prolif. 2021 Mar 3;54(4):e13012. doi: 10.1111/cpr.13012 (PMC8016644; doi:10.1111/cpr.13012)
Supplement: Supplementary file 1 — Supplementary Material [file CPR-54-e13012-s001.docx]

**Supplementary Figures**


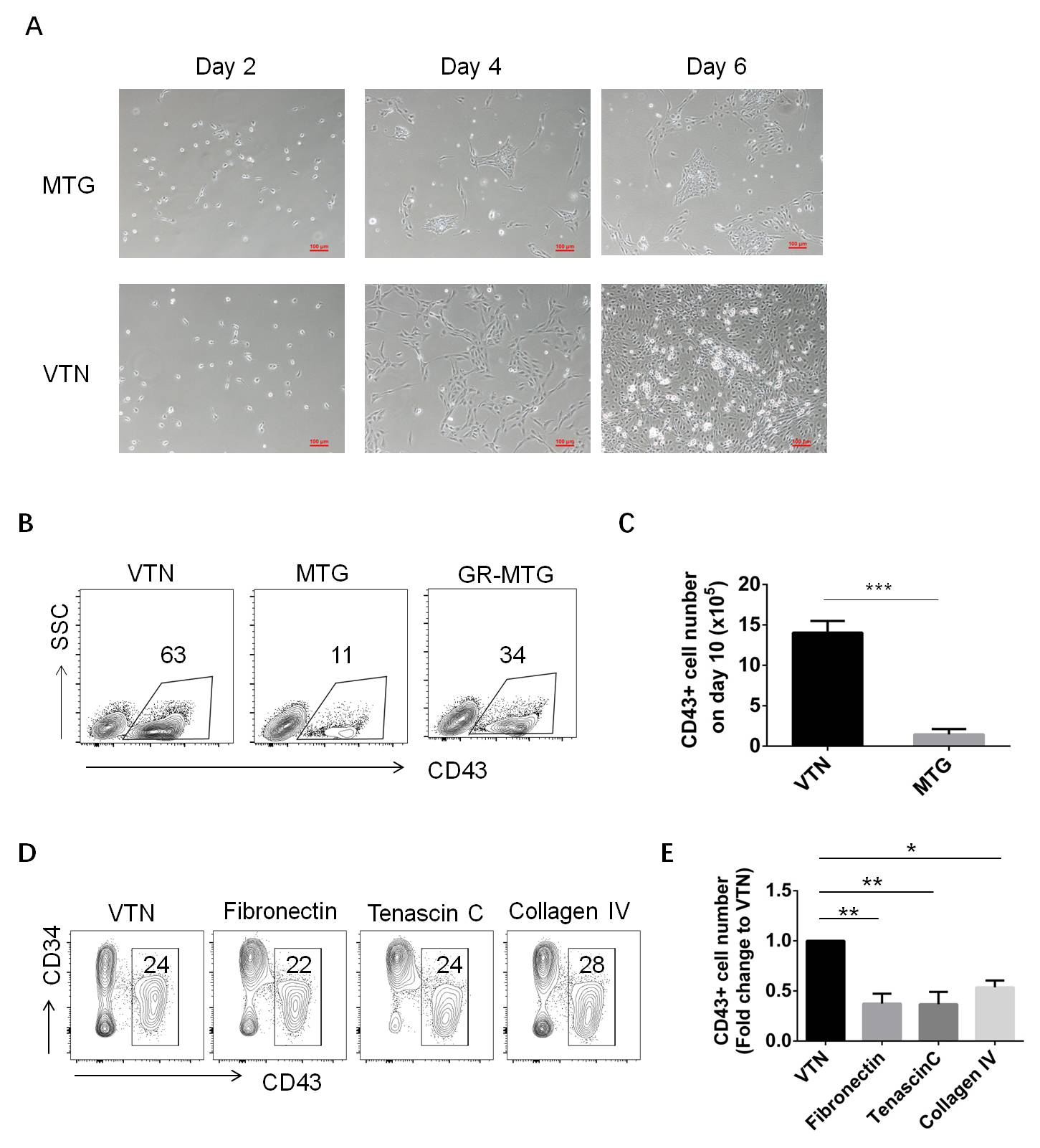


**Figure S1 VTN promotes early hematopoiesis of hPSCs, related to Figure 1.** (A) The representative images and typical morphology at different stages of hematopoietic differentiation in VTN or MTG-coated cultures. Scale bars, 100 µm. n = 3. (B) Flow cytometric analysis of the frequency of CD43+ HPCs generated in VTN, MTG or GR-MTG-coated cultures on day 8. n=3. (C) CD43+ cell number generated on day 10 in VTN- or MTG-coated cultures. n=6. (D) and (E) Flow cytometric analysis of the frequency and number (fold change) of CD43^+^ HPCs in the day 6 VTN (3μg/ml), or Fibronectin (3μg/ml), or Tenascin C (3μg/ml), or Collagen IV (3μg/ml)-coated cells. VTN was set as a control and normalized to 1. n=3.

**
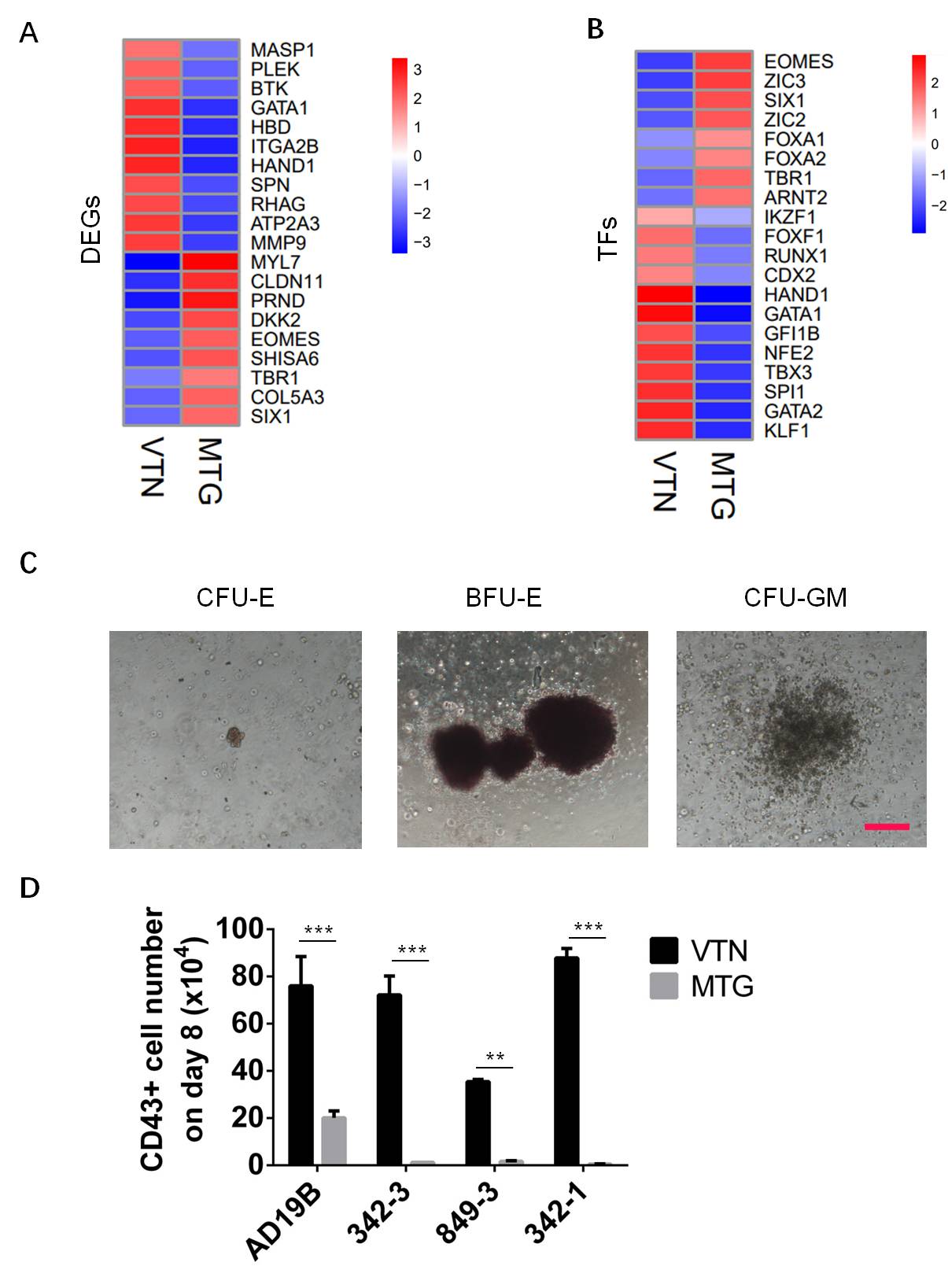
**

**Figure S2 hPSC-derived HPCs are enriched in semi-adherent cell clusters, related to Figure 2.** (A) Heatmap showing the expressions of top 20 DEGs between VTN and MTG. (B) Heatmap showing the expressions of top 20 transcription factors (TFs) between VTN and MTG. (C) Representative hematopoietic colony morphology generated from day 6 semi-adherent cells on VTN. Scale bars, 250 µm. (D) The number of AD19B, 342-3, 849-3 and 342-1-hiPSC-derived CD43+ cells on day 8 in VTN or MTG-coated cultures. n = 3.

**
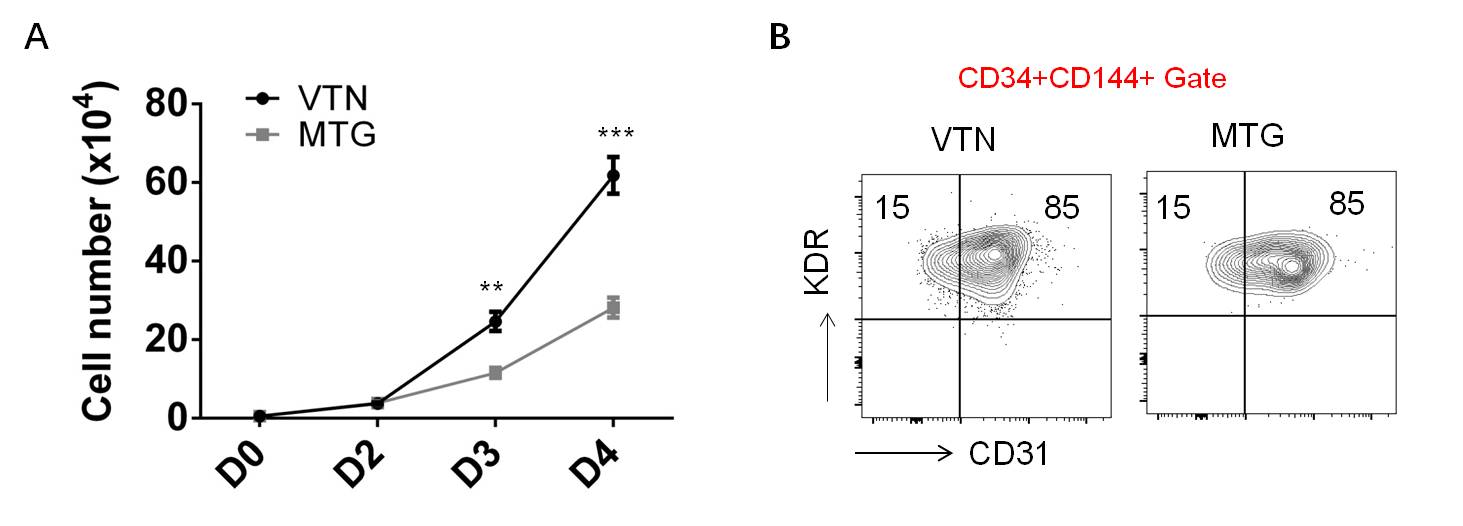
**

**Figure S3 VTN promotes the specification of hematopoietic-fated mesoderm and enhances HE generation from mesodermal progenitor cells, related to Figure 3.** (A)Total number of cells cultured in MTG or VTN at different time points. n=3. (B) Representative flow cytometric analysis of the CD31 and KDR expression in the day 4 CD34+CD144+ cells coated with VTN or MTG. n=3.

**
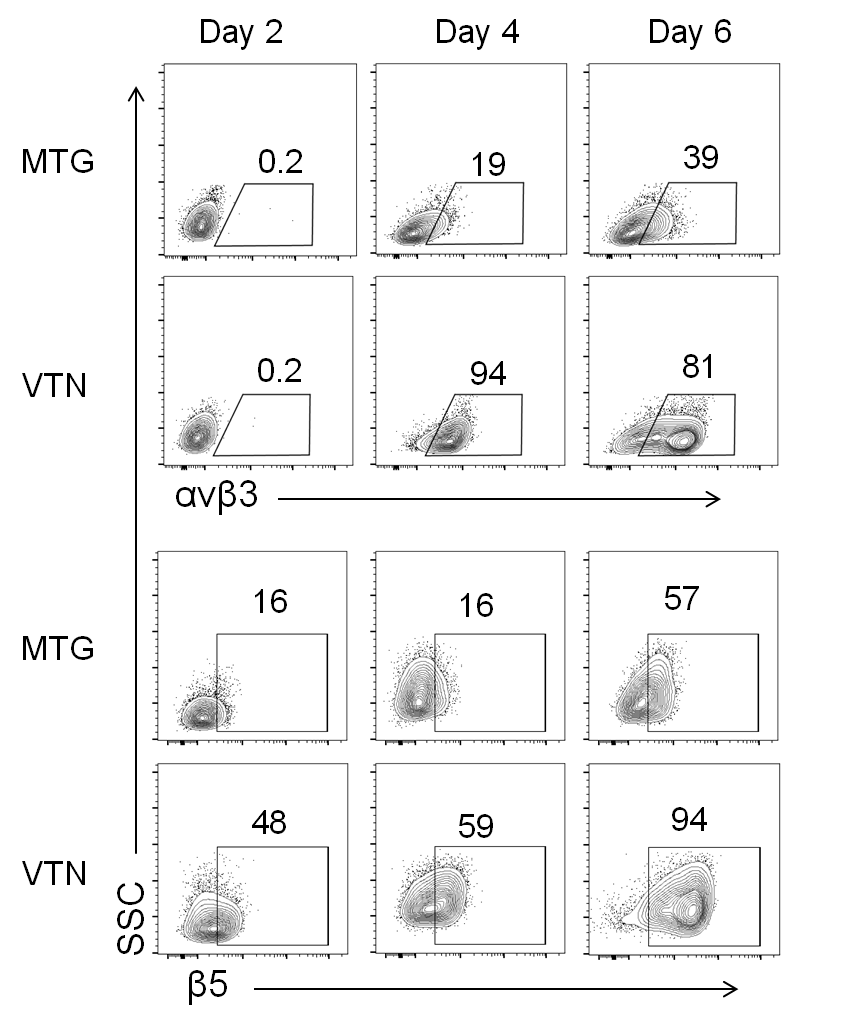
**

**Figure S4 The expression of αvβ3, as well as β5, was higher on VTN than that on MTG, related to Figure 4.** Representative flow cytometry analysis of the expression of αvβ3 and β5 integrins on day2, day 4 and day 6 in VTN or MTG-coated cultures. n=3.

**
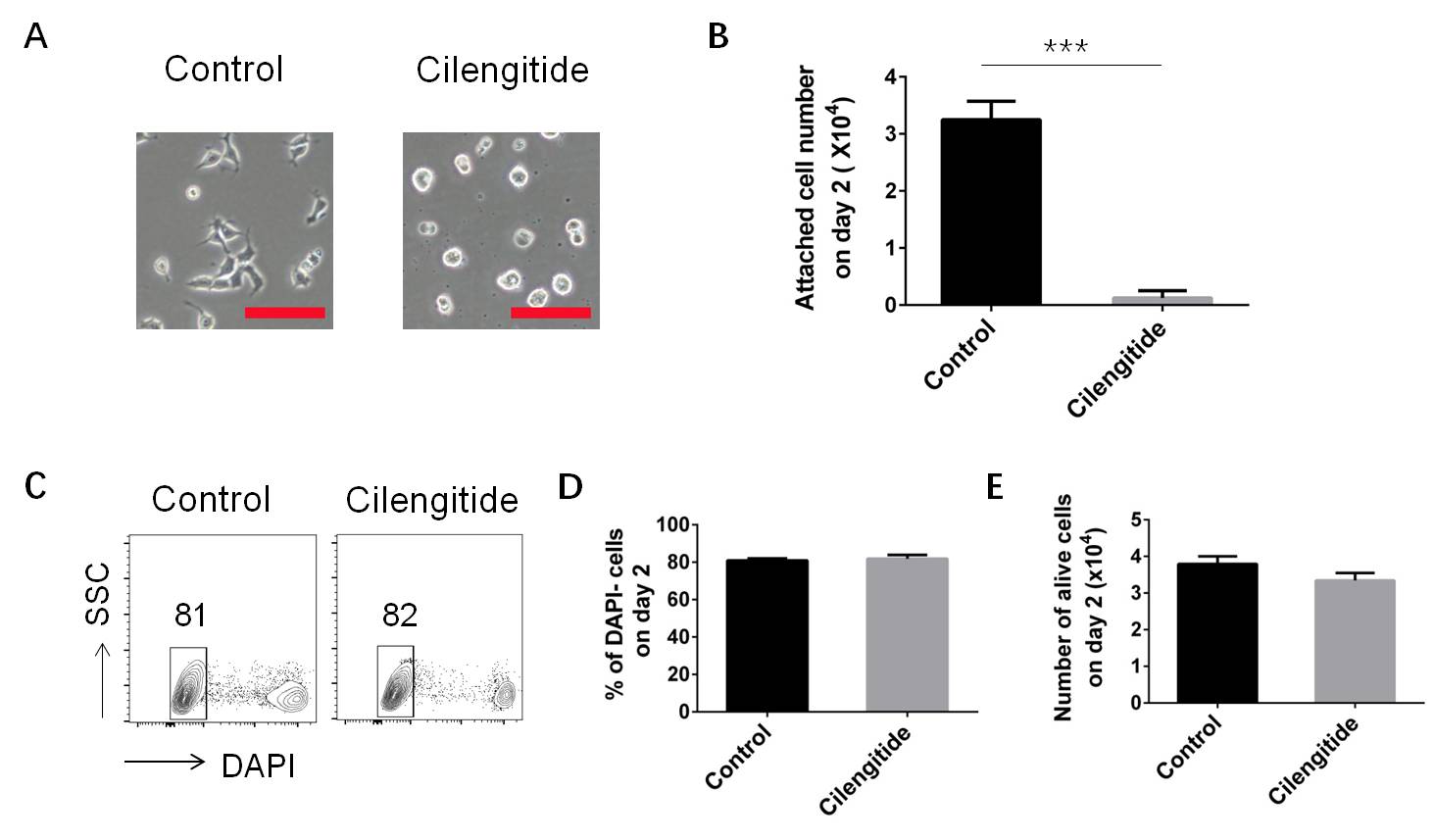
**

**Figure S5 Cliengitide treatment seriously impairs mesodermal cell adhesion, but do not affect cell survival and proliferation, related to Figure 4.** (A) Photomicrograph of the day 2 cells treated with or without Cilengitide. Scale bars, 50 µm. n=3. (B) The number of day 2 attached cells treated with or without Cilengitide. n=3. (C)-(E) Flow cytometric analysis of the frequency and number of day 2 DAPI^-^ alive cells treated with or without Cilengitide. n=3.

**
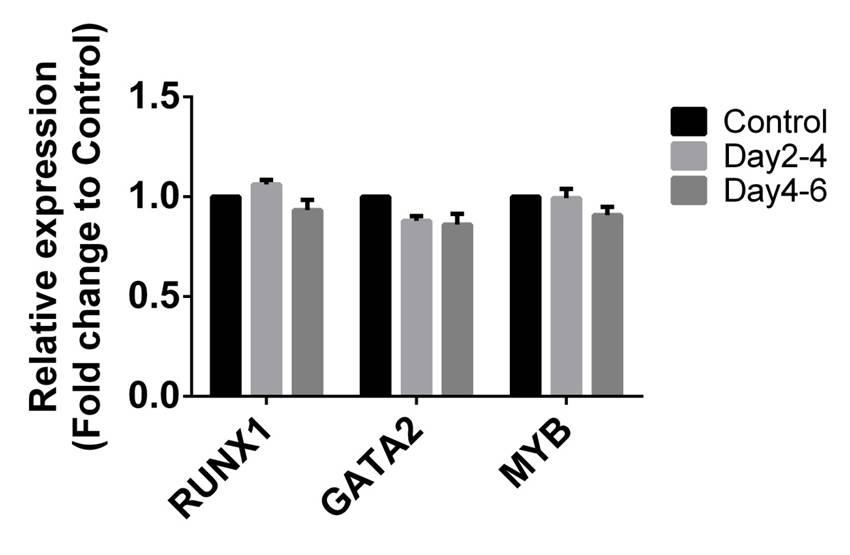
**

**Figure S6 ATN-161 does not impair hematopoietic development, related to Figure 5.** qRT-PCR analysis of *RUNX1*, *GATA2*,and *MYB* expression in the day 6 VTN-coated cells treated with or without ATN-161 between day 2 and day 4 (Day2-4) or day 4 and day 6 (Day4-6). n=3.


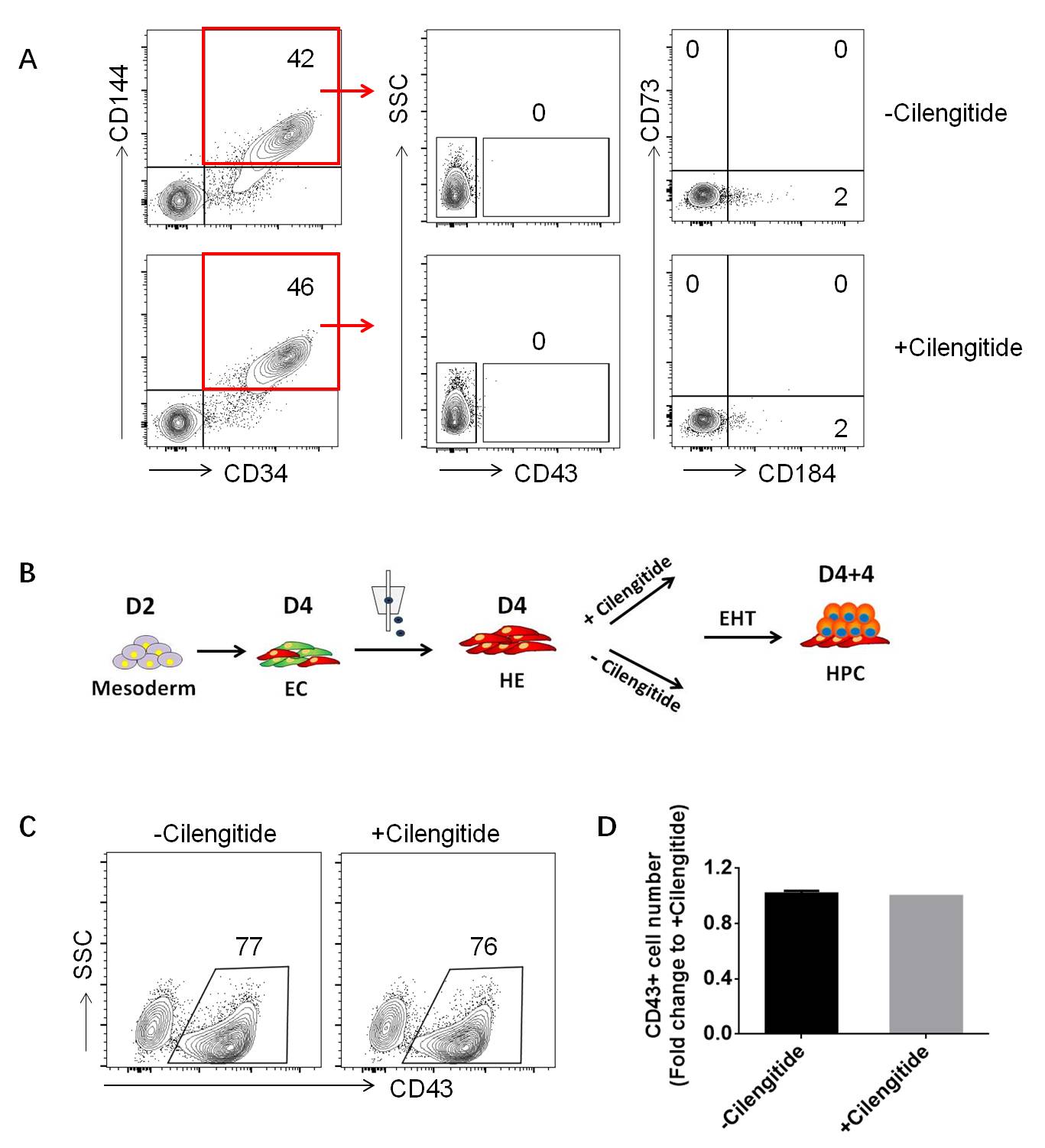


**Figure S7 αvβ3 and αvβ5 inhibition does not impair EHT, related to Figure 5.** (A) Representative flow cytometric analysis of the CD43,CD73 and CD184 expression in the day 4 CD34+CD144+ cells treated with or without Cilengitide. n=3. (B) Scheme depicting the strategy used for evaluating the effect of αvβ3 and αvβ5 on EHT. The day 4 CD34+CD144+CD43-CD73-CD184- cells were sorted and then re-seeded on VTN-coated plates for an additional 4 days EHT culture treated with or without Cilengitide for HPC generation. (C) and (D) The frequency and number of CD43+ cells generated from the day 4 CD34+CD144+CD43-CD73-CD184- cells following an additional 4 days EHT culture treated with or without Cilengitide. n=3.

**
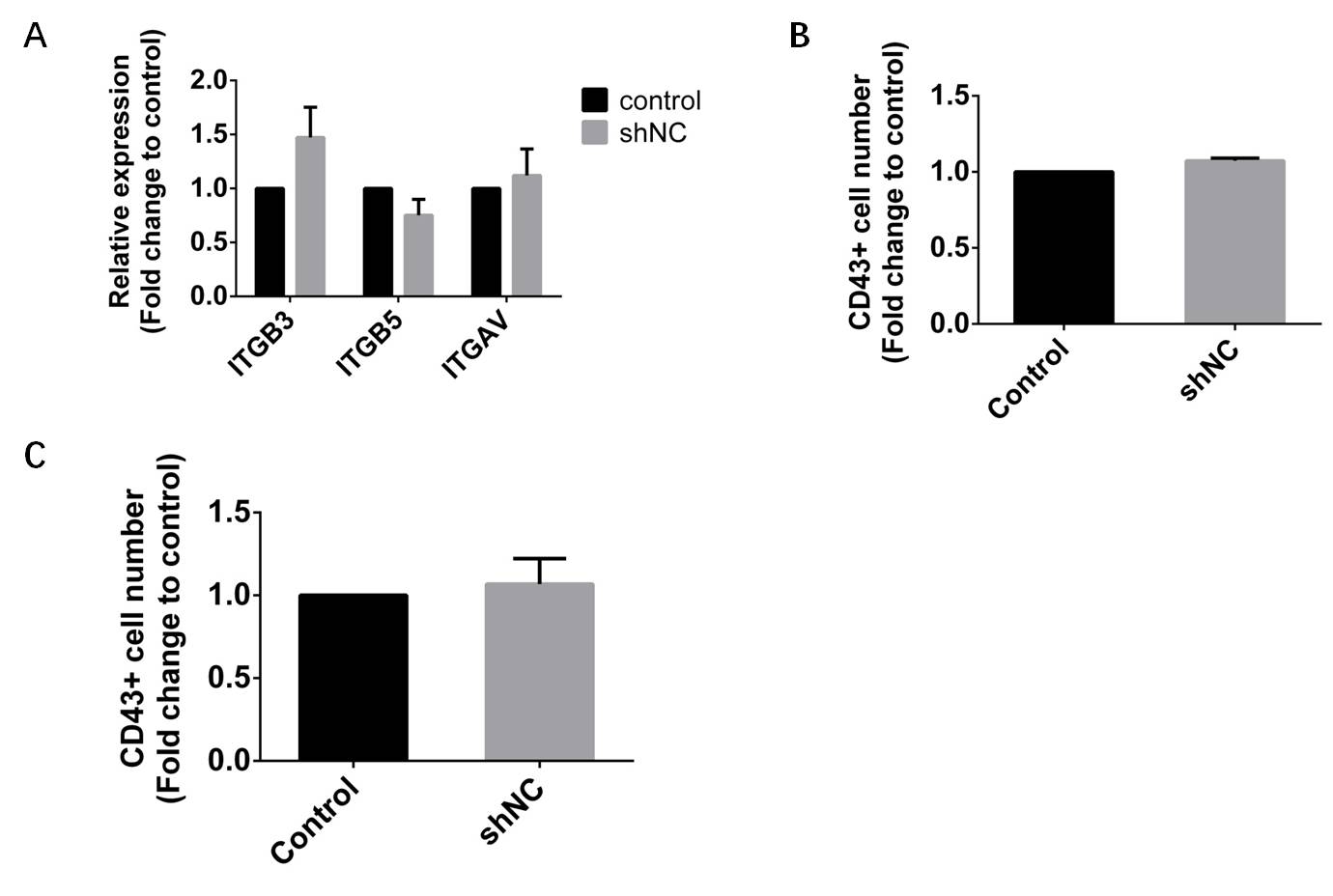
**

**Figure S8 Negative control shRNA (shNC) barely has any effect on neither *ITGAV*, *ITGB3* or *ITGB5* expression nor the production of CD43+ HPCs, related to Figure 6.** (A) qRT-PCR analysis of *ITGAV*, *ITGB3* and *ITGB5* expression in the day 6 VTN-coated cells treated with or without negative control shRNA (shNC) at Day2-4. Control group was normalized to 1. n=3. (B) The number of CD43+ cells generated in the day 6 VTN-coated cells treated with or without negative control shRNA (shNC) at Day2-4. Control group was normalized to 1. n=3. (C) The number of CD43+ cells generated in the day 6 VTN-coated cells treated with or without negative control shRNA (shNC) at Day4-6. Control group was normalized to 1. n=3.

**Supplementary table 1: Primers Used in This Study.**

| Gene | Forward Primer (5' -> 3') | Reverse Primer (5' -> 3') |
| --- | --- | --- |
| T | TATGAGCCTCGAATCCACATAGT | CCTCGTTCTGATAAGCAGTCAC |
| hMIXL1 | GGCGTCAGAGTGGGAAATCC | GGCAGGCAGTTCACATCTACC |
| TBX6 | CATCCACGAGAATTGTACCCG | AGCAATCCAGTTTAGGGGTGT |
| RUNX1 | CTGCCCATCGCTTTCAAGGT | GCCGAGTAGTTTTCATCATTGCC |
| MYB | CTGCAAAGTGAGGATTGGACG | CTTCAGGGCTCGAATGGCAT |
| GATA2 | GCAACCCCTACTATGCCAACC | CAGTGGCGTCTTGGAGAAG |
| ITGAV | GCTGTCGGAGATTTCAATGGT | TCTGCTCGCCAGTAAAATTGT |
| ITGB3 | GTGACCTGAAGGAGAATCTGC | CCGGAGTGCAATCCTCTGG |
| ITGB5 | AACTCGCGGAGGAGATGAG | GGTGCCGTGTAGGAGAAAGG |

Supplementary table 1: qRT-PCR primer sequences.

**Supplementary Video:**

**Time-lapse movie showing a typical budding process of EHT on VTN**

A typical budding process of EHT on VTN was captured by timelapse imaging revealing that a slender endothelium like cell gradually acquired round shape with CD43 expression (in red) and then gave rise to two semi-adherent round shape CD43+ hematopoietic cell. Scale bars, 20 μm
